# 1358. Establishing Genomic SARS-CoV-2 Surveillance at the National Level: Germany, 2021

**DOI:** 10.1093/ofid/ofac492.1187

**Published:** 2022-12-15

**Authors:** Djin-Ye Oh, Martin Hölzer, Sofia Paraskevopoulou, Maria Trofimova, Felix Hartkopf, Matthias Budt, Marianne Wedde, Hugues Richard, Berit Haldemann, Teresa Domaszewska, Janine Reiche, Kathrin Keeren, Aleksandar Radonić, Julia Patricia Ramos Calderón, Maureen Rebecca Smith, Annika Brinkmann, Kathrin Trappe, Oliver Drechsel, Kathleen Klaper, Sascha Hein, Eberhard Hildt, Walter Haas, Sébastien Calvignac-Spencer, Torsten Semmler, Ralf Dürrwald, Andrea Thürmer, Christian Drosten, Stephan Fuchs, Max von Kleist, Stefan Kröger, Thorsten Wolff

**Affiliations:** Robert Koch Institute (RKI), Berlin, Berlin, Germany; Robert Koch Institute (RKI), Berlin, Berlin, Germany; Robert Koch Institute (RKI), Berlin, Berlin, Germany; Robert Koch Institute (RKI), Berlin, Berlin, Germany; Robert Koch Institute (RKI), Berlin, Berlin, Germany; Robert Koch Institute (RKI), Berlin, Berlin, Germany; Robert Koch Institute (RKI), Berlin, Berlin, Germany; Robert Koch Institute (RKI), Berlin, Berlin, Germany; Robert Koch Institute (RKI), Berlin, Berlin, Germany; Robert Koch Institute (RKI), Berlin, Berlin, Germany; Robert Koch Institute (RKI), Berlin, Berlin, Germany; Robert Koch Institute (RKI), Berlin, Berlin, Germany; Robert Koch Institute (RKI), Berlin, Berlin, Germany; Robert Koch Institute (RKI), Berlin, Berlin, Germany; Robert Koch Institute (RKI), Berlin, Berlin, Germany; Robert Koch Institute (RKI), Berlin, Berlin, Germany; Robert Koch Institute (RKI), Berlin, Berlin, Germany; Robert Koch Institute (RKI), Berlin, Berlin, Germany; Robert Koch Institute (RKI), Berlin, Berlin, Germany; Paul Ehrlich Institute, Berlin, Berlin, Germany; Paul Ehrlich Institute, Berlin, Berlin, Germany; Robert Koch Institute (RKI), Berlin, Berlin, Germany; Robert Koch Institute (RKI), Berlin, Berlin, Germany; Robert Koch Institute (RKI), Berlin, Berlin, Germany; Robert Koch Institute (RKI), Berlin, Berlin, Germany; Robert Koch Institute (RKI), Berlin, Berlin, Germany; Institute of Virology, Charité-University Medicine Berlin, Berlin, Berlin, Germany; Robert Koch Institute (RKI), Berlin, Berlin, Germany; Robert Koch Institute (RKI), Berlin, Berlin, Germany; Robert Koch Institute (RKI), Berlin, Berlin, Germany; Robert Koch Institute (RKI), Berlin, Berlin, Germany

## Abstract

**Background:**

The COVID-19 pandemic has demonstrated the importance of pathogen genomic surveillance. At RKI, the German National Institute of Public Health, we established the Integrated Molecular Surveillance for SARS-CoV-2 (IMS-SC2) network to perform SARS-CoV-2 genomic surveillance.

**Methods:**

SARS-CoV-2 positive samples from laboratories distributed across Germany regularly undergo whole-genome sequencing at RKI. This surveillance instrument enables (i) almost-real-time monitoring of SARS-CoV-2 genomic diversity and evolution, (ii) *in vitro* assessment of vaccine coverage against emerging variants and (iii) genome-based estimates of SARS-CoV-2-incidences.

**Results:**

We report the results of our analyses of 3623 SARS-CoV-2 genomes collected between 12/1/2020 and 12/31/2021. All variants of concern were identified, at ratios equivalent to those in the 100-fold larger German GISAID sequence dataset from the same time period. Lineage distributions fluctuated over time, covering the rise of the Alpha and Delta, as well as the emergence of Omicron. Phylogenetic analysis confirmed variant assignments. Multiple mutations of concern emerged during the observation period. To model vaccine effectiveness *in vitro*, we employed authentic-virus neutralization assays, confirming that both the Beta and Zeta variants are capable of immune evasion. The IMS-SC2 sequence dataset facilitated an estimate of the SARS-CoV-2 incidence based on genetic evolution rates. Together with modelled vaccine efficacies, Delta-specific incidence estimation indicated that the German vaccination campaign contributed substantially to a deceleration of the nascent German Delta wave.

**Conclusion:**

This example illustrates that pathogen genomics enables a proactive approach to controlling a pandemic as the virus evolves. Molecular and genomic SARS-CoV-2 surveillance will be crucial during the post-pandemic future, informing public health policies including vaccination strategies. Of note, the IMS-SC2 infrastructure can be adapted to many other pathogens, serving as a blueprint for future efforts to increase genomic pathogen surveillance.

**Disclosures:**

**All Authors**: No reported disclosures.

